# E-Readers Are More Effective than Paper for Some with Dyslexia

**DOI:** 10.1371/journal.pone.0075634

**Published:** 2013-09-18

**Authors:** Matthew H. Schneps, Jenny M. Thomson, Chen Chen, Gerhard Sonnert, Marc Pomplun

**Affiliations:** 1 Science Education Department, Harvard-Smithsonian Center for Astrophysics, Cambridge, Massachusetts, United States of America; 2 Harvard Graduate School of Education, Harvard University, Cambridge, Massachusetts, United States of America; 3 Department of Computer Science, University of Massachusetts, Boston, Massachusetts, United States of America; University Children's Hospital Tuebingen, Germany

## Abstract

E-readers are fast rivaling print as a dominant method for reading. Because they offer accessibility options that are impossible in print, they are potentially beneficial for those with impairments, such as dyslexia. Yet, little is known about how the use of these devices influences reading in those who struggle. Here, we observe reading comprehension and speed in 103 high school students with dyslexia. Reading on paper was compared with reading on a small handheld e-reader device, formatted to display few words per line. We found that use of the device significantly improved speed and comprehension, when compared with traditional presentations on paper for specific subsets of these individuals: Those who struggled most with phoneme decoding or efficient sight word reading read more rapidly using the device, and those with limited VA Spans gained in comprehension. Prior eye tracking studies demonstrated that short lines facilitate reading in dyslexia, suggesting that it is the use of short lines (and not the device per se) that leads to the observed benefits. We propose that these findings may be understood as a consequence of visual attention deficits, in some with dyslexia, that make it difficult to allocate attention to uncrowded text near fixation, as the gaze advances during reading. Short lines ameliorate this by guiding attention to the uncrowded span.

## Introduction

Computers are beginning to transform how people interact with the written word. They have spurred an evolution in the social conventions for reading that is advancing at a rate arguably unprecedented in history. Importantly, computer-based technologies present options to reformat text in ways that are customized to the needs and preferences of the individual. In addition, they allow linkage to other tools (say, for search, notes, or accessibility) that enrich the reading process and otherwise broaden access. While some of such benefits are obvious and readily grasped, the impact of other advances is less clear, and their consequences for reading are as yet unknown. Because of the extraordinary pace of development, adoption, and changes in patterns of use, research has lagged the evolution in reading. Therefore very little is known about how the new approaches to reading will influence people's abilities to decipher the written word. Here, we expand on previous studies [1] to consider the effects of e-reader formatting on dyslexia. We investigate whether approaches enabled by these technologies can address the needs of those who currently struggle with reading on paper. Given that an estimated 5% to 17% of all readers face reading impairments due to the inherited neurological effects of dyslexia [2], the potential impact of such research can be substantial.

A number of investigators have previously proposed that adjustments in formatting or display of text may facilitate reading in dyslexia. Suggestions have included modifications to fonts [3], [4], rearrangements in page formatting [1], [5], [6], as well as a variety of methods to control the dynamics of reading [7], [8]. While, in some cases, benefits were noted, the effects were generally small and, occasionally, controversial and difficult to reproduce. One notable exception are findings demonstrating that increasing inter-letter spacing facilitates reading in children with dyslexia [9], presumably by counteracting an effect known as crowding that impairs object recognition in the presence of clutter [10], an effect observed to be more severe in many people with dyslexia [7], [9], [11]–[14].

Though prevailing models of dyslexia ascribe reading difficulties to poor phonological processing, in recent years dyslexia has been increasingly associated with deficits in visual attention (e.g., [15]–[24]) and poor oculomotor control [25]–[28], prompting a suggestion [5] that e-readers could be configured to reduce demands on visual attention and oculomotor control and thus make reading less of an effort for those impaired. A reading method called Span Limited Tactile Reinforcement (SLTR) was proposed, wherein text is displayed on a small screen handheld device (such as a smartphone), using large fonts so that the text spans only a few words per line. In the SLTR method, text is advanced by manually scrolling the text vertically, as if it were a long continuous column of newsprint.

In a previous experiment [1], we used gaze-tracking techniques to compare reading on a small screen e-reader (Apple iPod Touch) with reading on a larger tablet computer (Apple iPad), and found that, when students with dyslexia read using the iPod device, oculomotor performance markedly improved over reading using the larger format. In this paper, we extend this work and consider a direct test of the SLTR reading method, using methods that focus on comprehension, as opposed to the reading dynamics studied earlier. Here, we investigate the hypothesis that SLTR, implemented on a small screen handheld e-reader (Apple iPod Touch), is more effective than the traditional approach to reading using paper, for people who struggle with reading. We investigate this question in a cohort of 103 high school students with dyslexia, using reading comprehension and speed as dependent variables. Our results show that the hypothesis is partially supported: Those among the participants who have diminished VA Spans, or difficulties with phoneme decoding or sight word processing, benefit most from the SLTR method. Together, these findings suggest that this reading method is potentially an effective intervention for struggling readers. We discuss these results in the context of deficits for visual attention and crowding, and propose a possible explanation that includes an account for the high incidence of regressive saccades in dyslexia.

## Methods

### Ethics Statement

This study was approved by the Committee on the Use of Human Subjects in Research at Harvard University. In accordance with this, volunteers who were not minors provided written informed consent, while those who were minors provided written assent, with written consent additionally obtained from their parents or guardians.

### Research Design

**Figure 1:**
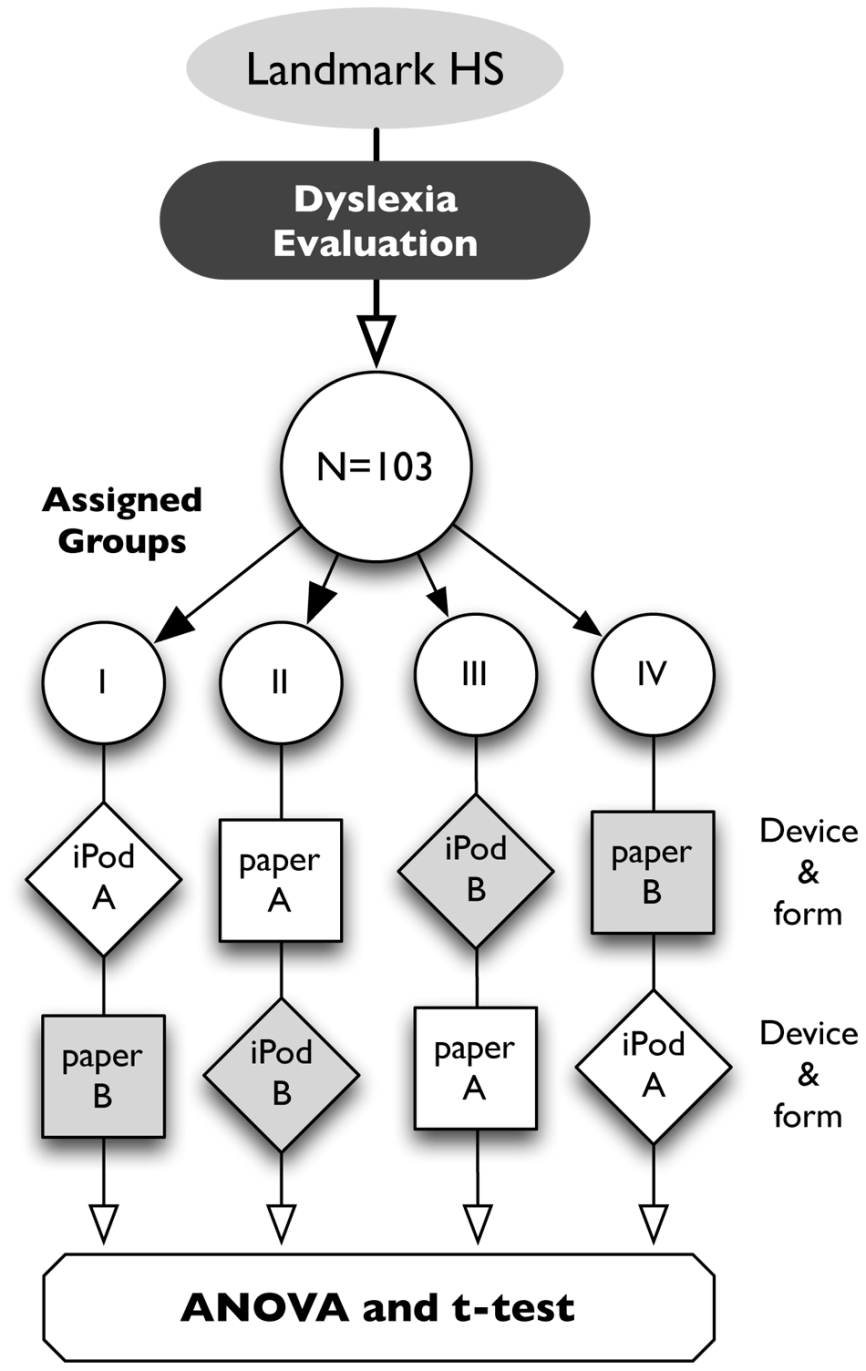
Research design. A cohort of 103 participants with dyslexia were assigned to four groups (I…IV), and each read two test Forms (A, B) from the Gates-MacGinitie Reading Tests [30] using paper or iPod, in a design balanced for order of device and form. The process was repeated at two Levels (7, 10).

The experimental design investigated the hypothesis that, among those with dyslexia, reading comprehension and speed would be greater with SLTR than when using the traditional method of reading on paper. Given, therefore, that our intent was to investigate the effects of a specific treatment on people with dyslexia, following accepted conventions in investigations of this sort (e.g., [29]), we employed a balanced within-subjects design, wherein participants served as their own controls. Thus, the two conditions (paper, iPod) were compared in a design of a “repeated measures” type, in which all subjects were measured in all conditions. The participants were subdivided into four randomly assigned groups (I, …, IV). Students read text from the Gates-MacGinitie Reading Tests [30], Levels 7 and 10, Forms A and B. As shown in the schematic (Fig. 1), each group read these two Forms of test materials (A, B) in a design that controlled for potential effects of presentation order (e.g., learning, fatigue, boredom) and of the specific text (e.g., Form A being less or more difficult than Form B), while examining differences by method (paper vs. iPod). The time taken to read the material was recorded, and comprehension was gauged using multiple choice questions associated with the text. Participants were tested using reading materials at two levels (7, 10). They were first tested at Level 7 using a set of Forms (A, B) targeting middle school readers. This was followed by a test at Level 10, using another set of Forms (A, B) targeting high school readers.

### Participants

Participants were 103 (64 male and 39 female) high school students with lifelong histories of reading struggles. All but one were enrolled at Landmark High School in Prides Crossing, MA (USA), a school exclusively for students with language-based disabilities. All students had vision that was normal or corrected to normal, and no histories of neurological disorders other than dyslexia. The current literacy and phonological awareness profile of each participant was measured prior to the experiment using the Test of Word Reading Efficiency (TOWRE; [31]), and three subtests of the Comprehensive Test of Phonological Processing (CTOPP; [32]). Participants' non-verbal ability was measured using the Block Design subtest of the Wechsler Abbreviated Scale of Intelligence; WASI [33]. The resulting characteristics of the cohort are summarized in Table 1. In addition, VA Span was observed using an adaptation (see below) of the 6-letter global report method described in [34], [35].

**Table 1:** Demographic statistics and reading measures of participants.

Variable	Obs	Mean	Std.	Min	Max
Grade	101	10.54	1.13	8	12
Years in Landmark	101	3.84	2.30	1	11
Gender (0 = male; 1 = female)	101	0.36	0.48	0	1
Age in years	101	17.09	1.29	14	19
Degrees of Reading Power Level*	101	59.27	13.95	26	98
***Standardized Scores*** *:* Block Design	81	47.38	10.23	12	68
Elision	103	8.91	2.18	3	12
Memory for Digits	103	9.15	2.99	2	16
Rapid Letter Naming	103	6.93	2.34	1	15
Rapid Digit Naming	103	7.58	2.36	2	15
Sight Word Efficiency	103	78.52	9.86	54	113
Phoneme Decoding Efficiency	103	79.71	8.26	60	100

*Supplied by school.

### Apparatus

#### (a) Paper

**Figure 2:**
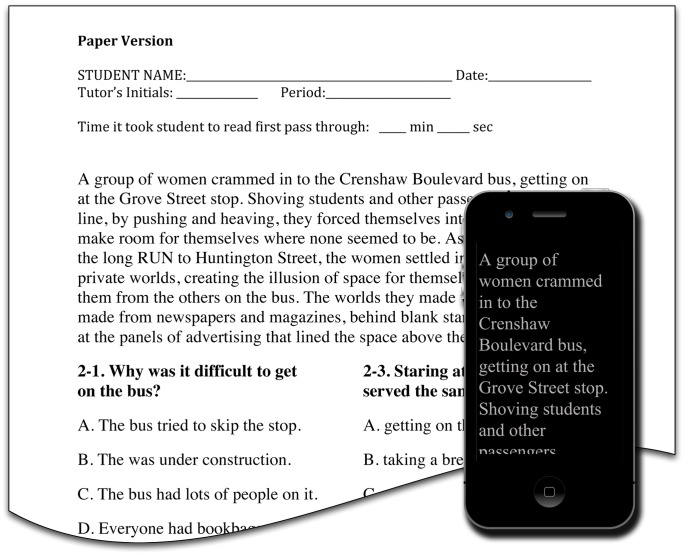
Sample stimuli comparing paper and iPod conditions. In the paper condition students read passages from the Gates-MacGinitie Reading Tests and answered multiple choice questions as shown. In the iPod condition the reading passage was displayed on the iPod (scrolled vertically using a finger on the touchscreen) and questions were answered on paper, as in the paper condition, except that the text passage was not displayed. Following standard protocol for this test, students were allowed to re-examine the text when answering questions, in both conditions.

In the paper condition, text was printed on normal white paper of dimensions 8.5×11 inches, using a 14 pt Times font, with 1-inch margins and normal single line spacing, with right-ragged margins, displaying an average of 13.94 (SD = 1.79) words per line (see Fig. 2). No formatting (such as bolding or italics) was used for emphasis, other than capitalization or use of normal punctuation. The paper was typically placed on a table, but was sometimes held in the hand during reading, at whatever distance was normal and comfortable for the participant (typically 35–45 cm). Ambient lighting conditions varied, and were typical for the classrooms in the school.

#### (b) iPod

Reading material prepared for the iPod condition was preloaded on an unmodified third generation Apple iPod Touch. The device had a screen resolution of 640×960 pixels at 128 pixels per cm. The luminance was set to a black level of approximately 0.9 cd/m^2^ and a white level of 66 cd/m^2^. Ambient lighting conditions varied, and were typical for the classrooms in the school. SLTR was implemented using the GoodReader app v.3.14.1 (http://www.goodiware.com). Here, text was displayed using a Times New Roman font at a setting of 42 pt, such that the distance between the tallest ascender and the lowest descender in the font measured approximately 0.65 cm. Thus, assuming the iPod was held at a nominal distance of 35 cm, a typical 8-character word would subtend about 3.6°. The text was left justified, with a right-ragged margin, and default line spacing was used, as illustrated in Fig. 2 (inset). The lines were short, displaying on average 3.40 (SD = 0.91) words per line. No formatting (such as bolding or italics) was included, other than capitalization or use of normal punctuation. To enlarge the effective dimensions of the margin the background was set to black (see Fig. 2), and the text was displayed as gray, at 75% of full white. The device was held freely in the hand, at a comfortable reading distance (typically about 35 cm), and text was scrolled as continuous stream, manually advancing the text in a vertical direction using a touch gesture on the screen.

### Stimuli

For purposes of the experiment, reading material from [30] was excerpted and consistently reformatted (without their accompanying illustrations) for the paper and iPod conditions. Each Form (A, B) and each Level (7, 10) contained twelve reading passages. The 12 reading passages successively increased in length and complexity. Level 10 was more difficult than Level 7, but within each Level, Forms A and B were designed to be of comparable difficulty and differ only in content. The passages were read either on paper or on the iPod, but in both cases participants responded on paper to multiple-choice questions pertaining to the passage read.

### Procedures

To control for potential bias due to novelty, students practiced reading using SLTR on the iPod for a minimum of 300 minutes prior to testing, typically in sessions that were 30 minutes long, distributed over a period of 10 days. Here, students chose practice materials from among a number of popular age-appropriate e-books offered to them. The reading practice was monitored for fidelity by a proctor from the school. Students read silently, and the proctor periodically engaged the students in conversations about the material read, and asked questions about its content. The fidelity of practice was subjectively scored and recorded. Following practice reading, testing was carried out in four 45-minute sessions. Students were allowed to reread the text while answering questions. Reading speed was measured using a stopwatch. The same procedures were followed for each of two Levels (7, 10). Following standard protocol for the Gates-MacGinitie test, testing was stopped at 35 minutes, and the tests were scored using the instructions provided with the test.

### VA Span

A 6-letter global letter report task was used to measure VA Span (“visual attention span”) using custom software (iCue) on the iPod device, adapting procedures described in [35]. The participant held the iPod in the hand, at a comfortable reading distance. The participant manually started each trial by tapping the device touchscreen. This initiated a 1000 ms presentation of a number (1–10) centrally placed on the screen that the participant read aloud (used to facilitate score-keeping). Following this, a blank screen appeared for 1000 ms, and then a centrally placed fixation marker, held for 1000 ms. Fixation was followed by a second blank screen of 500 ms duration. A five-letter global report task followed immediately, wherein 6 unique characters, each separated by four spaces, were chosen with no order constraint from among (B, P, T, F, L, M, D, S, R, H), and displayed on screen for 200 ms using a 20 pt fixed-width Courier font. The string of letters spanned 4 cm on the display. The letter string presented in each trial was unique. Following the global report stimulus, a blank screen appeared, at which point participants reported any letters recalled, irrespective of order, with no constraint on time. Following a practice session, 24 trials were presented. The number of correctly identified letters was totaled to create a score.

## Results

Hierarchical linear models with student as the grouping variable were estimated for two dependent variables: reading comprehension score and reading speed. The comprehension score is the number of items answered correctly on a 48-item test (i.e., the highest achievable EES score was 48; the overall mean was 26.7 and the standard deviation was 10.0). Reading speed was measured in words per second (with an overall mean of 2.4 and a standard deviation of 1.2). The independent variables were method (1 = iPod; 0 =  paper), VA Span, SW, and PD. VA Span is the mean of the number of letters correctly reported in each trial of the VA Span task. The SW score is the standard score derived from the number of sight words correctly read in 45 seconds from the TOWRE. PD is the standard score derived from the number of nonwords correctly decoded in 45 seconds, also from the TOWRE. The models were adjusted for unequal variances between Level 7 and 10 by estimating the variances separately by level.

### Reading Comprehension

We estimated a main effects model of the reading comprehension score, a model that included all possible interactions between the predictors, and a final model that included only the significant effects. Table 2 shows the main effects model and the final model.

**Table 2:** Analysis of Reading Comprehension Score.

	Main effects model					Final model				
Effect	Estimate	Std. Err.	DF	t Value	p value	Estimate	Std. Err.	DF	t Value	p value
Intercept	2.49	6.54	201	0.38	0.7041	−3.91	5.51	217	−0.71	0.4785
method	−0.32	0.42	203	−0.75	0.4548	4.92	2.09	202	2.35	0.0196
PD	−0.10	0.09	200	−1.03	0.3044					
SW	0.34	0.08	200	4.41	**<.0001**	0.30	0.07	202	4.47	**<.0001**
VA Span	1.66	1.03	200	1.61	0.108	2.16	1.04	241	2.09	**0.038**
method*VA Span						−1.59	0.62	202	−2.55	**0.0114**

**Notes to** **Table 2:** N(subjects)  = 103; N(observations)  = 410.

**Figure 3:**
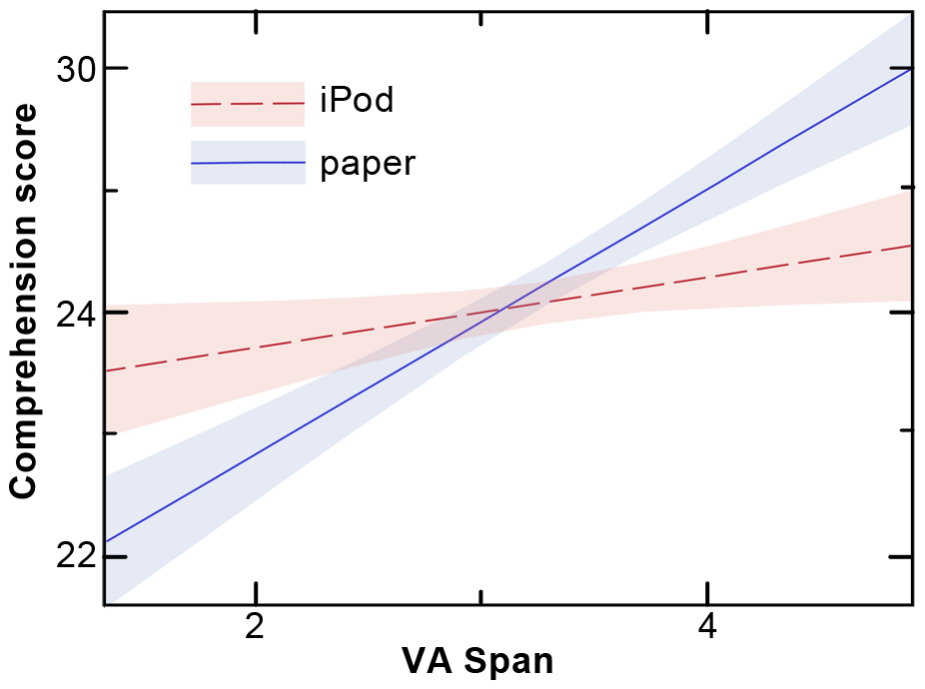
Shape of the interaction of method and VA Span for comprehension. A significant interaction of method*VA Span was observed when comprehension was taken as the dependent variable. Here, the mean comprehension score is shown as a function of VA Span, the number of letters correctly identified on a six letter global report paradigm. The iPod is indicated in red, and paper is indicated in blue. The figure shows that those with low scores on the global report task comprehend better when reading on the iPod while the reverse is true for those with high scores. (The colored shading indicates a confidence interval for this interaction, defined by a +/−1-sigma within-subjects standard error of the mean [68]).

There was a positive main effect of SW (higher SW values predicting higher comprehension scores; p<.0001) and an interaction between VA Span and method (p = 0.0114). This interaction indicated that iPod reading yielded higher scores than did paper reading for subjects with low VA Span, whereas paper reading was superior to iPod reading at high VA Span (see Fig. 3). (Note that the indicated significance for the method main effect in the final model is merely a statistical artifact of the interaction—the main effects model demonstrates that there is no main effect of method.)

### Reading Speed

**Figure 4:**
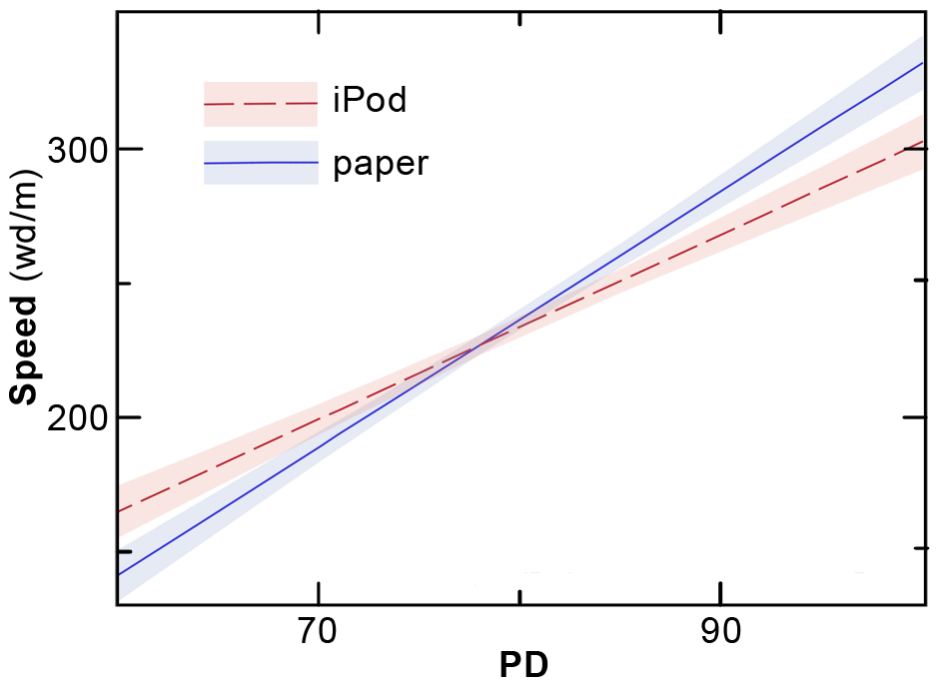
Shape of interaction of method and PD for reading speed. A significant interaction of method*PD was observed when reading speed was taken as the dependent variable. Here, the speed is shown as a function of PD, a measure of phonemic decoding. The interaction indicates that those with poor phonemic decoding scores perform better when reading on the iPod, while the reverse is the case for those with strong phonemic decoding scores. (Coloring, etc., as in Fig. 3.)

Again, we estimated both main effects and comprehensive interaction effects models. After eliminating non-significant effects, the following final model emerged. It included significant main effects of SW (p = 0.001) and PD (p = 0.0002); both variables were positively related to reading speed. Furthermore there was a significant interaction between PD and method (p = 0.0451). The shape of the interaction can be seen in Figure 4. It indicates that iPod reading held a speed advantage at low levels of PD, but not at high levels of PD where paper reading had the speed advantage.

## Discussion

### E-reader facilitates speed and comprehension for some and not others

Interactions dominated the observed effects in this experiment, such that some individuals read better using paper, while others read better via iPod. Due to the interaction, these effects averaged out when considering the sample as a whole, and significant main effects of method, favoring one method over the other, were not observed (see Tables 2 and 3). The observed interaction is such that those who had diminished VA Span scores were able to comprehend better when reading on the iPod than on paper. Likewise, considering reading speed, those who had poor phoneme decoding skills, or those with poor sight word reading skills (significant for Level 7 only) read faster using the iPod than reading on paper.

**Table 3:** Analysis of Reading speed.

	Main effects model					Final model				
Effect	Estimate	Std. Err.	DF	t Value	p value	Estimate	Std. Err.	DF	t Value	p value
Intercept	−3.21	0.75	196	−4.3	<.0001	−3.69	0.78	241	−4.74	<.0001
method	−0.02	0.06	191	−0.39	0.6984	1.04	0.53	188	1.97	0.0508
VA Span	0.02	0.12	195	0.21	0.8351					
SW	0.03	0.01	194	3.34	0**.001**	0.03	0.01	195	3.37	**0.0009**
PD	0.04	0.01	194	3.84	**0.0002**	0.05	0.01	232	4.45	**<.0001**
method*PD						−0.01	0.01	189	−2.02	**0.0451**

**Notes to** **Table 3:** N(subjects)  = 103; N(observations)  = 387. Whereas there was no significant interaction between method and SW in the full dataset, such an interaction was present when the analysis was restricted to only the level 7 texts.

### VA Span and Visual Attention

It is widely accepted that dyslexia results from difficulties associating orthographic and phonological information during reading [36], [37]. However, in recent years it has become increasingly evident that deficits in multimodal attention also act to impair low-level functionalities implicated in reading (e.g., [15]–[24]), a phenomenon readily apparent in transparent languages, such as Finnish or Italian, where phonological difficulties pose less of a confound. For example, a longitudinal study of Italian-speaking children showed that pre-reading impairments in visual attention predict dyslexia once children learn to read, implying a possible functional association of dyslexia and attention [18]. The extent to which these factors impede reading in opaque languages (such as English), where it is thought that deficits in phonological processing are predominantly implicated in dyslexia [38], is a topic actively debated in the field [39]. Nevertheless, given that visual attention deficits are associated with dyslexia in transparent orthographies, there is no reason that such deficits will not also factor, at least in some individuals, in languages that are opaque, to act either in concert with, or independent of, deficits for phonological processing [40]. In our study, we find that the VA Span task serves to distinguish those who benefit from iPod formatting and those who do not, and it is likely that it is deficits in visual attention that are being characterized and tapped by the VA Span task [41] to give rise to the interactions we observe (Fig. 3).

The global report version of the VA Span task used in this experiment [35] concurrently taps into a number of processes important in reading. Several authors stress a distinction between systems for focal attention and those for rapid distributed spatial attention that act in concert to build the visual percept in a complex scene [42], [43]. The global report task briefly flashes a string of widely-spaced letters at fixation and scores the number identified, and thus taps into abilities to distribute attention over a span of about 4°. Given that the letter string is briefly flashed while the gaze is held at fixation, focal attention directed to individual letters acts concurrently with distributed attention in this task. Furthermore, by requiring participants to recall these letters, the task also invokes processes for working memory [44], and naming [45]. And because no backward mask is used, iconic visual memory plays a role as well. However, given that the VA Span task presents letters that are widely spaced, crowding is not expected to factor significantly. Nor is phonological processing thought to be important in this task, as behavioral [46] and imaging [34] studies suggest that response to the VA Span task is dissociated from such processes. Thus, the fact that those with poor performance on the global report are observed in our study to comprehend better when using the iPod than paper suggests that SLTR is serving to address deficits related to distributed attention, working memory, or both, through mechanisms that are as yet unknown.

### Phonemic Decoding and Sight Word Reading

Phonological decoding and sight word reading are observed to interact with reading method, when reading speed is considered in our experiment. The measures of PD and SW used here capture accuracy and speed concurrently (e.g., number of words correctly read in 45 seconds). Thus, these parameters are indicative of capacities for rapid word recognition that facilitate automaticity. It is therefore, perhaps, not surprising that formatting manipulations facilitating those who lack automaticity, impedes those who are skilled. After all, text formatting normally used in reading is socially engineered to be maximally efficient, and thus highly effective for those who are typical readers. Efficient readers are able to accurately control the dynamics of their gaze and make use of the long lines of text, by advancing fixations efficiently along the string. Reading with SLTR likely slows typical readers, as now the text must be moved manually, instead of relying on the gaze alone. In contrast, our study suggests that those who are less adept at such automatic processes benefit by reading on the device, likely because of oculomotor effects, as we discuss this in more detail below.

We emphasize that our study was carried out in a sample of high school students with dyslexia who had received extensive remediation, averaging 3.84 years (see Table 1) of targeted immersive instruction at the Landmark School. Therefore, a possible alternate explanation of the finding that students with poor phoneme decoding and/or sight word reading skills benefit more from SLTR is that these students may simply have started from a position of diminished reading ability, perhaps because they had less exposure to remediation, or because they had performed poorly in these classes, and therefore had more to gain. However, when the variable “years spent at Landmark” was included in the analysis, no significant correlations of interest were revealed, arguing against this hypothesis.

#### iPod Improves Oculomotor Efficiency

Dyslexia has been associated with oculomotor deficits that include erratic fixation and poor saccadic control, and those with dyslexia are observed to make more and longer fixations and more frequent regressive saccades during reading [26]–[28], [47]–[50]. The fact that SLTR displays text in a narrow window using large fonts may serve to diminish demands for positional accuracy of fixation, and thus perhaps provide advantages in the iPod condition. Furthermore, because SLTR text is manually scrolled with the finger, the demands for gaze tracking are also reduced. These considerations are in contrast to the paper condition, where readers must accurately control their gaze and track gaze positions along relatively long lines.

The suggestion that formatting used in the iPod display helps readers with dyslexia manage oculomotor demands is supported by findings from a previous experiment performed in our laboratories [1]. Here, eye-tracking methods were used to observe reading in 26 high school students with dyslexia. This experiment compared reading on an iPod with reading using a larger tablet computer (Apple iPad). Unlike the current study, SLTR was not used to display and advance the text in this experiment. Instead, the text on the iPod was formatted to display 2.19 words per line (similar to line lengths used here) and compared this with an iPad display formatted for 11.6 words per line (comparable to 13.94 words per line in the paper condition in the present study). Other than this, the text formatting used for both iPod and iPad was similar in both conditions. Eye tracking revealed that the iPod condition was strongly advantageous: reading speed was enhanced by 27% in the iPod condition, without loss in comprehension. Also, consistent with the present findings, the eye tracking study found that those who struggled most with reading benefited more from the iPod treatment. Notably, readers made fewer fixations overall, and inefficient gaze movements made to re-inspect words were reduced by a factor of two when the iPod was used.

**Use of iPod as an intervention for struggling readers.** Our study demonstrates that reading using short lines, displayed via small handheld e-readers, improves reading comprehension and speed in some readers with dyslexia. All of the 103 high school students participating in this study have faced lifelong struggles with reading that have been sufficiently debilitating to warrant enrollment (often at great cost to families, local school systems, or both) in a special school focused on reading intervention. Given that those in this sample attended these programs for a minimum of 1 to 11 years (mean 3.84; SD 2.30), these participants represent a highly compensated sample. The gains in reading comprehension we observe are therefore over and above those accrued as a result of intensive remediation offered by the participants' school, suggesting that, for some people, reading using such devices can provide a boost to their reading comprehension that adds to whatever gains are made through traditional approaches.

Within this special sample, what proportion of students stood to benefit from the iPod intervention? Examining the interaction shown in Fig. 3, those with VA Span scores below 3.09 showed advantages in comprehension reading with the iPod, compared with reading on paper. Likewise, those with PD scores below 77.8 showed gains in speed from iPod reading (see Fig. 4). Examining the distribution of scores within our sample, a third (32.4%) of the participants have VA Span scores below this cutoff, and hence are expected to benefit from iPod reading. For PD, almost half (45.6%) are below the cutoff and hence are expected to benefit. Thus, even though we cannot extrapolate to the general population from these findings obtained in a special sample of highly compensated high-school students, we can nevertheless conclude that the iPod method is beneficial for substantial numbers of students, when used as an adjunct to more traditional reading interventions and support.

**Proposed mechanisms. **Why does the iPod formatting improve reading in those who are most impaired? While this study cannot directly address this question, insights from our prior eye tracking study [1] and the literature on the gaze dynamics of reading provide important clues. Though the discussion that follows in this section is clearly speculative, we offer these ideas to stimulate hypotheses that motivate future research.

### Short lines facilitate reading by guiding attention to the uncrowded span

Models for reading, such as the E-Z Reader model [51] and SWIFT [52], have been proposed to explain how low-level visual processes, such as visual attention, interact with higher-level cognitive processes, such as linguistic analysis, to drive eye movements during reading. These models generally assume that an “attentional spotlight” directs attention forward in text [15], to selectively process a span of characters during brief instances of fixation that occur between rapid shifts in the gaze during reading. In this context, visual attention deficits in dyslexia can be postulated to slow reading by (1) diminishing the extent of the perceptual span used in reading [35], [46], and by (2) impeding the advance of this span by slowing engagement and disengagement of attention as the gaze moves [21], [23], [53].

However, the processes described above are not the only ways attention interacts with eye movements to regulate the dynamics of reading. Crowding, a neurological phenomenon that impairs peripheral recognition of flanked characters, fundamentally limits the number of letters that can be perceived at a glance [10]. Therefore, in order to read efficiently, attention must be directed to the uncrowded span of text, centered at fixation [54], as the gaze shifts during reading. We suggest that attention deficits associated with dyslexia make this a challenge by disrupting processes needed to maintain attention to the uncrowded span, as fixations advance from one word to the next [1].

This possibility is corroborated by a case study of an individual with “selective attentional dyslexia” [55]. In this study, a gaze-contingent display was used to admit a window of text yoked to fixation. Either random letters or X's masked the text outside this window. When random letters masked text outside a span of 15 characters centered on fixation, the individual with dyslexia read poorly compared with the controls. However, when X's were used to create a similar mask, this individual (who was otherwise a very poor reader) remarkably read as if unimpaired, at rates comparable to the natural reading speeds of typical readers. We interpret this to suggest that, when X's were used as the mask, the gaze-contingent display served to guide attention to the span of uncrowded text centered at fixation, and thus to ameliorate deficits in attention. However, when random letters were used as the mask, the boundary demarcating the span of uncrowded text was indistinguishable from normally crowded text, and the person with attentional dyslexia (otherwise lacking clues to guide attention to the uncrowded span) read very poorly. We suggest that, in the case of our experiments, the use of short lines in the iPod condition similarly helps those with visual attention deficits by guiding attention to a narrow window of the text, which facilitates allocation of attention to the uncrowded span as the gaze shifts in reading.

### Short lines help inhibit perception of text previously read

**Figure 5:**
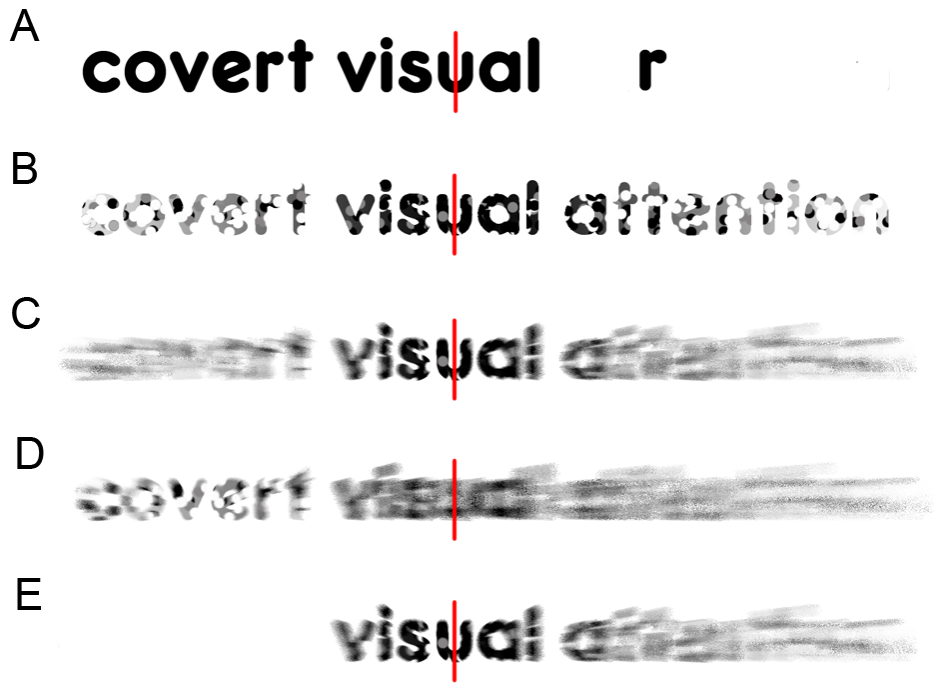
A proposed explanation: Short lines guide attention to the uncrowded span. (A) Crowding is easily demonstrated. Fixating on the red line, most of the characters in the word “visual” can be identified, while those in the adjacent word “covert” (say, the letter “r”), viewed peripherally, are difficult to discern. However, when peripheral letters are viewed in isolation (the “r” to the right), the uncluttered text is more readily identified. This peripheral interaction phenomenon is referred to as crowding [67]. (B) Crowding increases with angle from fixation, as suggested schematically by the stippling. Given this, only the word closest to fixation (“visual”) falls within the “uncrowded span” [10] that is easily read. (C) Therefore, as the gaze shifts during reading, attention (here suggested using a radial blur) must track the uncrowded span as fixations advance. (D) However, for those with attention deficits, attention shifting is sluggish [53], and we suggest that this causes attention to be slow to disengage from the previously fixated word (“covert”) as the gaze advances. Attention is therefore over-emphasized in the periphery (left of fixation), where words are subject to crowding and difficult to discern. We propose that these factors conspire to make reading difficult in some people with attentional forms of dyslexia. (E) Short lines ameliorate such deficits by guiding attention to the uncrowded span, while minimizing confusion caused by the presence of crowded text to the left of fixation.

Our prior eye tracking study [1] showed that, when reading long and short lines of text (11.6 versus 2.19 words per line) was compared in students with dyslexia, the incidence of regressive saccades decreased by a factor of two when short lines were used. Because the decreased line length was not accompanied by an expected trade-off in the incidence of horizontal and vertical regressions, this indicated that the use of the narrow formats affected phenomena local to the fixated word, perhaps to control misperception of crowded words located to the left of fixation. This finding is consistent with the hypothesis that the use of short lines in the iPod condition controls regression simply by eliminating crowded text to the left that would otherwise drive attention away from the uncrowded span and promote inefficiencies during reading (see Fig. 5).

Under conditions of normal reading, readers allocate attention to a perceptual span that is asymmetric, extending a few letters to the left of fixation, but as many as 15 letters toward the right [56]–[58]. Despite this, typical readers also attend to text located to the left, at sites previously attended during reading [59]–[61]. For example, when gaze-contingent displays are used to substitute words skipped during reading, people regress to these altered words more often when the substitution introduces a conflict with expected meaning, demonstrating that people also attend to text left of fixation even when this text was never read [60]. While typical readers are able to inhibit perception of such text to the left, visual attention deficits may make it difficult for some with dyslexia to do so.

Oversensitivity to text to the left of fixation could arise as a consequence of sluggish attention shifting in dyslexia [17], [21], [23], [53], [62]. Here, sluggish attention shifting is presumed to slow the rate at which attention disengages from previously fixated words. Therefore, as fixations advance from one word to the next, attention spreads to the left and overemphasizes perception of crowded text in fields previously read. Perception of this text can introduce confusion, and in cases cause a regressive saccade to be issued to clarify meaning [1]. A strong test of this hypothesis would use gaze-contingent displays to examine attention shifts during a sequence of saccades made through an array of targets and distractors (cf., [63]) to compare the balance in attention between the previous location and the next location, regardless of absolute performance. Comparing response in dyslexia and typical readers, we would expect the bias toward the previous location to be greater in dyslexia than in the typical readers.

## Conclusions

We find that SLTR on a small-screen handheld device facilitates reading by improving both speed and comprehension in a subset of high school students with dyslexia. This supports and expands on emerging work, demonstrating that relatively simple adjustments to the visual presentation of text, in this case shortening the lines, or in other experiments adding spacing between letters and lines to control crowding [1], [9], [11], can facilitate reading in those who struggle, or in at least some of them. The findings here support and complement conclusions of prior eye tracking research [1], and those studies are consistent with our interpretation that gains result primarily from the use of shortened lines, which serve to moderate inefficient gaze motions in reading. Future investigations might focus on how the dynamic allocation of attention interacts with eye movements and crowding during reading, as formatting can influence such mechanisms, controlled through use of popular e-reader devices.

While reformatting the page significantly improves reading in those with dyslexia, we emphasize that this alone cannot address all of the factors known to impede reading. Altering spatial formatting can only partially alleviate factors affecting the temporal dynamics in reading, such as slowness caused by sluggish attention shifting [17], difficulties accessing phonological representations of words [37], latencies in naming [45], or difficulties with character recognition [64], [65], each of which can act, independently of the effects addressed here, to additionally impair reading. Furthermore, given that people's reading characteristics vary [66], it is reasonable to expect that the benefits of reformatting the page will likely vary between individuals, as observed here. In support of this, we found that those with smaller VA Spans, poor phonological decoding skills, and diminished sight word reading benefit from SLTR presentations, whereas those who are stronger in these areas do not.

In the century since dyslexia was first described, methods used for reading have undergone very little change. However, with the widespread adoption of e-readers and other digital technologies for reading, reading methods are rapidly evolving, opening the possibility that alternate methods for reading can perhaps reverse historically imposed constraints that have caused so many to struggle, and make reading accessible to many currently excluded.
